# Beliefs regarding COVID-19 vaccinations of young adults in the United Kingdom: An interview study applying the Integrated Change Model

**DOI:** 10.1371/journal.pone.0277109

**Published:** 2022-12-06

**Authors:** Ilja van Bergen, Sophie Böger, Charlotte Beaudart, Mickaël Hiligsmann, Kei Long Cheung

**Affiliations:** 1 Faculty of Health, Medicine and Life Sciences, Maastricht University, Maastricht, The Netherlands; 2 Health Behaviour Change Research Group, Department of Health Sciences, College of Health Medicine and Life Sciences, Brunel University London, London, United Kingdom; 3 Department of Health Services Research, Care and Public Health Research Institute (CAPHRI), Faculty of Health, Medicine and Life Sciences, Maastricht University, Maastricht, The Netherlands; The University of the West Indies, TRINIDAD AND TOBAGO

## Abstract

**Introduction:**

Young adults are considered one of the most hesitant groups towards getting vaccinated in the UK, which threatens the success of the vaccination program in ending the pandemic. Identifying and understanding the socio-cognitive beliefs is important to effectively design and implement health communication interventions. Therefore, the aim of this study was to identify the underlying beliefs regarding COVID-19 vaccinations among young adults in the UK.

**Methods:**

The study consisted of online, one-on-one interviews with 18 individuals (6 males, 12 females) aged between 18 and 29 years, conducted in June 2021. The guiding theoretical framework was the I-Change Model. Interviews were recorded and transcribed verbatim. The transcripts were independently coded by two researchers by using the constructs of the I-Change Model. Belief statements were elicited from the codes and the frequency of belief statements was recorded and compared between intenders and non-intenders.

**Results:**

Similar beliefs were observed in intenders and non-intenders for most constructs of the I-Change Model. However, non-intenders distinguished themselves from intenders by their higher perceived risks of side effects and higher perceived disadvantages of being vaccinated. Non-intenders expressed the belief that the risk of unknown or long-term side effects, such as blood clotting and impact on fertility, were the main reason for them not to be willing to vaccinate. In addition, in both groups, participants had mostly similar beliefs as their friends and family.

**Conclusion:**

This research provides insights in the specific beliefs of the young adult population of the UK regarding COVID-19 vaccinations, which could have implications for health communication interventions. The findings suggest that such interventions should focus on reducing the uncertainty regarding short- and long-term effects and potentially having a focus on the entire social environment of young adults.

## Introduction

On January 30th, 2020, the World Health Organization (WHO) declared the outbreak of the novel coronavirus (SARS-CoV-2, referred to as COVID-19) a global public health emergency [1]. Symptoms of an infection with the virus can vary from mild illness including sore throat, headaches, chills to severe or fatal illness [2]. In the United Kingdom (UK), to date (18^th^ July, 2021), 5,433,939 people have been diagnosed with the novel coronavirus, of which 128,708 people have died within 28 days after diagnosis [3]. As the primary route of transmission is through respiratory droplets from face-to-face contact, contaminated surfaces or aerosols, governments had to implement measures such as movement restrictions, mandatory face masks, closure of public spaces, and social isolation. As a result, the COVID-19 outbreak did not only put pressure on the healthcare system, it also has large socio-economic consequences [4] which forced countries worldwide to be highly resilient.

Vaccines are believed to be the best solution to be able to return to the pre-pandemic patterns of working and socializing. As a result, there has been a global explosion of vaccine development and new vaccines were developed rapidly [5]. In December 2020, the UK became the first country to approve one of the COVID-19 vaccines for emergency use [6]. At the time of this study, four different vaccines based on two different platforms were available in the UK: the mRNA-based vaccines by BionTech/Pfizer (BNT162b2) and Moderna (mRNA-1273) and the vector-based vaccines by Oxford/AstraZeneca (AZD1222) and Janssen (Ad26.COV2.S) [7]. After the first vaccines were approved, vaccination quickly became the foremost global defence strategy in combating COVID-19 [8].

However, not only do the vaccines have to be available, vaccines also have to be accepted by the health community and the general public in order to be effective in stopping the virus from spreading [9]. Due to the highly infectious nature of COVID-19, it is important that a large proportion of the population gets vaccinated or the chain of transmission to be broken. However, as stated by Umakanthan and Lawrence [10], there is a growing body of evidence that vaccine hesitancy is increasing while at the same time vaccination rates are decreasing in many countries.

Vaccine hesitancy is also a topic of concern in the UK. Previous research has shown that, before the vaccines became available, 72% of the population was likely to accept a vaccine [11]. Even though the positive sentiment has increased ever since, with 94% of the adults reporting a positive sentiment between 28 April and 23 May 2021, the young adult population reported the largest percentage (13.0%) of vaccine hesitancy among all age groups [12]. The same findings emerged from another study, which concluded that younger age is significantly associated with vaccine hesitancy in the UK [13]. Low vaccination rates are concerning, since the coverage threshold to achieve herd immunity is estimated to be between 55.0% and 82.0% of a population. Moreover, certain population groups may not be eligible for vaccination because of their age or pre-existing medical conditions [9]. In addition, vaccinations are never perfectly efficacious, meaning that vaccination levels cannot be considered equal to immunity levels [14]. Resultant, a vaccine refusal rate that is 10.0% or larger can significantly reduce the success of vaccination programs [9].

Due to the relatively high percentage of vaccine hesitancy among young adults in the UK, it is of high importance to specifically target this population group when designing health communication interventions. The importance of targeting young adults is further stressed by the current infection rate patterns, which demonstrate a significantly higher prevalence of infections in younger age groups than in older age groups. It was therefore concluded that vaccinating those aged 18 and older could substantially reduce the spread of the virus [15]. Furthermore, it was demonstrated by Chen and Orenstein [13] that enthusiasm for new vaccines is usually highest prior to and directly after their release. According to the UK prioritising system for the vaccine distribution, young adults were the last population group to become eligible, meaning that their enthusiasm for vaccinations may have significantly reduced. This could imply that there is a higher vaccination resistance rate among young adults than numbers were previously showing.

In order to stimulate vaccination uptake, health communication interventions are in use. However, the relatively high percentage of hesitant young adults demonstrates that current interventions are suboptimal. According to the Intervention Mapping approach, health communication interventions are most effective if they incorporate the relevant determinants of behaviour change and target those beliefs specifically [15]. Moreover, as described by Haldane et al. [16], community engagement is crucial for a system to be resilient in response to a crisis. Thus, in order to optimise the effectiveness of these targeted or tailored health communication programs, it is paramount to understand the specific socio-cognitive beliefs of the target population [15]. This is done by comparing intending and non-intending individuals by applying the Integrated Change Model (I-Change Model), following the pragmatic approach by Cheung et al. [17]. Hence, the aim of this study is to explore the relevant socio-cognitive determinants of vaccination behaviour among young adults in the UK which are potentially important for behaviour change.

## Methods

### Interview topic guide

Interviews were held based on a pre-established topic guide. The first part of the guide consisted of demographic questions regarding age, gender, education, employment status, residence and vaccination status. Then, questions regarding beliefs on COVID-19 vaccinations followed. The questions for this part were guided by the I-Change Model, and were developed while integrating the model with the guidelines of Atkins et al. [22] in applying the Theoretical Domains Framework (TDF) of behaviour change. The I-Change Model is widely used in research about the determinants of health behaviour and the uptake of health interventions. The model integrates the ideas of the Social Cognitive Theory, the Health Belief Model, Theory of Planned Behaviour, the Trans-Theoretical Model, and Goal setting theory. The underlying mechanism behind behaviour change according to the I-Change Model, is that behaviour change occurs through three different phases: the awareness, motivation, and action phase. Each of the phases has its relevant determinants. For the awareness phase, the relevant determinants are *knowledge* and *risk perception*. The relevant determinants for the motivation phase are *attitude*, indicated by advantages and disadvantages, *social influence*, and *self-efficacy*. For the action phase, the relevant determinants are *action planning*, *preparatory planning* and *coping planning* [17].

The purpose of the topic guide was twofold: 1) to elicit belief statements about each construct of the model and 2) to compare the underlying beliefs of individuals intending and not intending to vaccinate. Specific questions were developed using other studies applying the I-Change Model as well as studies regarding intention to vaccinate and vaccine hesitancy [18–20]. Questions were open-ended and participants were encouraged to elaborate freely on questions and to provide a certain amount of details and explanations where needed, as recommended by Polit & Beck [21]. Before the start of the study, three pilot interviews were conducted. Following the pilots, the interview guide was reviewed and minor amendments were applied in order to ensure clarity, interview length and coverage of constructs. Furthermore, prompts were added to ensure that all constructs were addressed. The final interview guide can be found in S1 File.

### Sampling and participants

The online participant recruitment tool *Prolific* was used for recruitment. The *Prolific* tool for recruitment was chosen to be able to recruit a sample of the target population within a short period of time. Moser and Korstjens [22] guidelines for data saturation in content analysis were applied. Inclusion criteria applied to the Prolific database were age (18–29 years old), UK residency, and willingness to participate in a video-call interview. Participants of the pilot interviews were excluded to avoid double participation. After the first ten interviews, a preliminary analysis of the data was conducted. Resultant from the recruitment strategy, ten participants all intending to vaccinate were included in the sample. As the intention of this research was to compare intending and non-intending individuals and considering the time frame of the study, it was deemed necessary to extend the sample by purposively recruiting individuals not intending to vaccinate. Therefore, in a second recruitment round, the screener ‘COVID-19 vaccine opinions–against (I feel negative about the vaccines)’ was added. Participation to the study was entirely voluntary, and participants received a monetary reward proportionate of their time investment, in accordance with *Prolific’s* requirements of paying participants.

### Procedure

Participants were approached via *Prolific* and received the participant information sheet, with a link to the online booking survey in Google Forms. Within the same survey, the participants had to fill out questions regarding their informed consent and to confirm that they were willing to participate in the study. The participants finally had to provide their anonymous *Prolific* ID numbers, allowing the researchers to arrange the logistics of the interviews through the anonymous *Prolific* messaging system.

All interviews were conducted online, using the Microsoft Teams software for video-calls. Besides the interviewer, one moderator was present. Microsoft PowerPoint slides designed for this study were used to guide the interview. Participants were explained the purpose of the study, after which they were asked to again confirm their informed consent and give permission to record the interview. Each interview lasted approximately 20 minutes. The recordings of the interviews were transcribed verbatim and responses were anonymised. After transcription, the recordings were destroyed.

### Analysis

In order to facilitate the analysis, an abductive theoretical perspective was used, meaning a mix of deductive and inductive approaches was applied to facilitate the content analysis [23]. The deductive approach was applied by using the theoretical constructs of the I-Change Model as the starting point for the coding guide. The three pilot interviews were coded independently by two coders into the constructs of the I-Change Model and themes were identified from the participants’ quotes. The coding of the two researchers was compared to develop the final coding guide. All following interviews were also independently coded by using Nvivo (released in March, 2020).

During the coding process, the coders compared interviews and used an iterative process to correct differences and ensure consistency. Moreover, themes that reoccurred multiple times but were not part of the original guide were added as sub-constructs to the existing constructs of the I-Change Model, fulfilling the inductive component of the analysis as described by Timmermans & Tavory [23]. When multiple constructs could be identified within one response, the entire response was coded in all constructs to avoid losing context by splitting up responses [24]. Simple percentage agreement/disagreement was applied to calculate the coding reliability and to assess consistency of coding. When both coders coded the same utterance in the same construct, complete agreement was achieved [25]. A total of 721 utterances from the 18 interviews was coded into 9 constructs including 16 sub-constructs. Initial simple percentage agreement between the independent coders across all interviews and constructs was 78.5%. Coding differences were resolved by consensus discussion.

After coding, one researcher generated statements representative of beliefs of the respondents, following the strategy as applied by Patey et al. [26]. Specific beliefs are statements providing detail about the perception of participants on the identified themes present in the constructs of the I-Change Model. Reoccurring themes were coded as multiple instances of the same belief and summarized into statements representing the utterances with the same meaning [26]. The generated statements were reviewed by the aforementioned research team to ensure the content was represented in an accurate manner. The frequency of each belief was recorded; in each interview, each belief was counted once.

### Ethics

Ethical approval was obtained from the Brunel Research Ethics Committee (30437-A- Jun/2021-32844-1) and the Maastricht University Research Ethics Committee (FHML/HPIM/2021.008). Informed consent was obtained from all participants by including questions in the booking survey as well as verbal confirmation at the start of the interview.

## Results

### Description of the sample

The sample (n = 18, referred to as *total*) consisted of twelve females and six males, aged 18–29 years old, living in nine different regions of the UK. Recruitment took place between 14^th^ and 18^th^ of June 2021. Participants varied in their highest level of education completed as well as their employment status. Thirteen participants reported to not been vaccinated yet, of which five did not intend to vaccinate at all. The remaining eight unvaccinated participants reported that they had their invitation and apart from two, all had booked a vaccination appointment. Four participants reported to have received one dose and one participant reported to be fully vaccinated at the time of the interview. A detailed overview of the sample can be found in Table 1. People intending to vaccinate are further referred to as *intenders* (n = 13) and people not intending to vaccinated as *non-intenders (*n = 5).

**Table 1:** Summary of demographic characteristics.

		*total*	*intenders (n-13)*	*non-intenders (n = 5)*
***Age (years)***	*Median*	*26*	*25*	*28*
	*Mean (SD)*	*25*.*3*	*24*.*5*	*27*.*4*
***Gender (%)***	*Male*	*6 (33*.*3)*	*6 (46*.*2)*	*0 (0*.*0)*
	*Female*	*12 (66*.*7)*	*7 (53*.*8)*	*5 (100*.*0)*
***Region (%)***	*Wales*	*1 (5*.*6)*	*1 (7*.*7)*	*0 (0*.*0)*
	*Greater London*	*4 (22*.*2)*	*3 (23*.*1)*	*1 (20*.*0)*
	*Scotland*	*3 (16*.*7)*	*2 (15*.*4)*	*1 (20*.*0)*
	*South West*	*1 (5*.*6)*	*1 (7*.*7)*	*0 (0*.*0)*
	*South East*	*3 (16*.*7)*	*2 (15*.*4)*	*1 (20*.*0)*
	*West Midlands*	*2 (11*.*1)*	*2 (15*.*4)*	*1 (20*.*0)*
	*East Midlands*	*2 (11*.*1)*	*1 (7*.*7)*	*1 (20*.*0)*
	*Northern Ireland*	*1 (5*.*6)*	*0 (0*.*0)*	*1 (20*.*0)*
	*Yorkshire*	*1 (5*.*6)*	*1 (7*.*7)*	*0 (0*.*0)*
***Education (%)***	*A levels*	*4 (22*.*2)*	*4 (30*.*8)*	*0 (0*.*0)*
	*Degree*, *Bachelor*	*9 (50*.*0)*	*5 (38*.*5)*	*4 (80*.*0)*
	*Degree*, *Master*	*5 (27*.*8)*	*4 (30*.*8)*	*1 (20*.*0)*
***Employment (%)***	*Student*	*5 (27*.*8)*	*5 (38*.*5)*	*0 (0*.*0)*
	*Unemployed*	*1 (5*.*6)*	*1 (7*.*7)*	*0 (0*.*0)*
	*Employed*, *part-time*	*3 (16*.*7)*	*0 (0*.*0)*	*3 (60*.*0)*
	*Employed*, *full-time*	*9 (50*.*0)*	*7 (53*.*8)*	*2 (40*.*0)*
***Vaccination status (%)***	*Unvaccinated*	*13 (72*.*2)*	*8 (61*.*5)*	*5 (100*.*0)*
	*Partially vaccinated (1 dose)*	*4 (22*.*2)*	*4 (30*.*8)*	*0 (0*.*0)*
	*Fully vaccinated*	*1 (5*.*6)*	*1 (7*.*7)*	*0 (0*.*0)*

### Awareness phase beliefs

Table 2 presents the observed beliefs of the awareness phase. Participants were asked what they knew about the COVID-19 vaccinations in terms of purpose, eligibility, safety and manufacturers. Statements were checked for correctness and no major misconceptions were observed in both groups. Participants had a generally equal, rather superficial understanding of the purpose of COVID-19 vaccinations. Most participants (88.9% of total) were able to explain the priority system, name the manufacturers (77.8% of total) and explain how vaccinations work (83.3% of total). Their main source of information was the news (61.5% of intenders and 60% of non-intenders). Other sources mentioned were scientific articles, social media, or friends and family working in the field of vaccinations and health.

**Table 2:** Awareness phase beliefs.

Construct and sub-themes	Specific belief	Sample quotes	Frequency intenders N (%) (n = 13)	Frequency non-intenders N (%) (n = 5)
**Knowledge (%)**				
	Aware of the different vaccine manufacturers	“Yes, so Astra Zeneca, Pfizer, Moderna. I know Johnson and Johnson (…) which is American and Sputnik which is the Russian one, and there is a Chinese one but I can’t remember what’s that called exactly”	10 (76.9)	4 (80.0)
	Aware of the priority system	“It’s been done in phases” “I know that the government has this kind of like hierarchy. Like a top-down list of most vulnerable to least vulnerable.”	12 (92.3)	4 (80.0)
	Aware of what (COVID-19) vaccinations do	“They tend to either stop you from getting a disease or tech your body how to fight the disease” “To learn to defend you from that whatever it is the vaccine for”	11 (84.6)	4 (80.0)
	Aware of the safety requirements of COVID-19 vaccinations	“Other vaccines usually take longer (…), so that’s one of the reasons why I’m aware of it being faster than historically other vaccines have been”	5 (38.5)	1 (20.0)
	Aware of the requirement of two doses	“All the ones that are available in the UK are two vaccinations”	3 (23.1)	1 (20.0)
	News websites as the main source of information	“Mainly the news, so like BBC or the Guardian or the Telegraph, mainly online” “News, like, what comes up on BBC, Apple News”	8 (61.5)	3 (60.0)
	Aware of the blood clotting controversy	“I know there has been some controversy, I believe about Astra Zeneca, because of the risk of blood clotting”	4 (30.8)	3 (60.0)
	Knowledge about effectiveness rates	“I know that the Pfizer one supposed to be like 90%, then some of the others around 70 or 80%”	5 (38.5)	0 (0.0)
**Risk perception (%)**				
*Susceptibility (COVID-19)*	Likely to catch COVID-19 at this moment	“Prior to vaccination I think it’s very likely”	4 (30.8)	0 (0.0)
	Unlikely to catch COVID-19 at this moment	“Very unlikely” “Not very likely”	9 (69.2)	5 (100.0)
	Likely to catch COVID-19 because the infection rate is high.	“Because especially with the cases going back up again now I think people are wearing less masks now, so I think it’s likely”	3 (23.1)	0 (0.0)
	Unlikely to catch COVID-19 because the infection rate is low.	“The city I live in is a not a very high infection zone, so I believe my risk of contracting is quite low” “The risk itself would be quite minor for myself, because the rates of COVID-19 are falling in the UK”	3 (23.1)	0 (0.0)
	Likely to catch COVID-19 because of meeting people	“I have been on the underground while it was busy, and people are wearing less masks now, so I think it’s likely” “I think it is likely because I have been in social situations recently”	5 (38.5)	0 (0.0)
	Reduced risk of catching COVID-19 after vaccination	“I think without the vaccine I would have been likely” “I believe after I get vaccinated, my risk of contracting it falls even more”	8 (61.5)	0 (0.0)
	Unlikely to catch COVID-19 because of not catching it before	“I think I just haven’t had it just far, I probably stopped wearing the mask about six months ago or more, I haven’t contracted COVID (…) I just don’t think it’s very likely at this point”	2 (15.4)	3 (60.0)
	Unlikely to catch COVID-19 because of taking the necessary precautions	“When I do go out I wear a mask, sanitize my hands, just taking all sorts of precautions to reduce their spreads” “I am careful and I’m not always like going out a lot, but I don’t think that means I’m immune to it”	3 (23.1)	1 (20.0)
*Severity of COVID-19*	Perceived consequences of a COVID-19 infection are mild or non-existent symptoms	“depending on my age, I am not so at risk” “I think for someone with my age (…), you just catch COVID and you have a severe flu and you stay at home for 10 days or until you feel better” “I don’t have any of the underlying symptoms that could cause complications”	9 (69.2)	4 (80.0)
	Uncertain about the impact of a COVID-19 infection on personal health	“I can’t say it’s not affecting me or if it’s going to affect me or not” “I think it’s quite unknown because it could either be just like there are no symptoms, or I could have a cold or a fever, but then you don’t know if you’re going to have a severe reaction to it”	3 (23.1)	1 (20.0)
	High perceived likelihood of getting seriously ill after a COVID-19 infection	“But if I got COVID, I can lose my life” “I suppose it could get serious and I might need the hospital”	6 (46.2)	1 (20.0)
	Potentially experiencing long-term consequences of a COVID-19 infection.	“I’m definitely aware of or more concerned about the long term impacts because I know there has been some studies that have looked at it and I know that some people have reported reduced lung capacity for quite a while afterwards”	3 (23.1)	0 (0.0)
	Reduced likelihood of severe illness due to COVID-19 after being vaccinated	“in case you do catch it, … the consequences could be a lot worse for you …” “Now I’ve already had a small dosage of a similar thing, (…), the risk is dramatically lower”	3 (23.1)	0 (0.0)
	A COVID-19 infection negatively affects quality of life	“I mean like the quality of life could definitely be”	1 (7.7)	0 (0.0)
	Unable to go to work due to a COVID-19 infection	“(…) and I have to come out of work so I won’t be very helpful there”	0 (0.0)	2 (40.0)
	Likely to infect other people when being infected with COVID-19	“The main risk would be passing it on to other people (…), I would be concerned about passing it to my parents and other older people”	1 (7.7)	0 (0.0)
*Risk of side effects*	Side effects of COVID-19 vaccinations are mild	“Some people have experienced sort of flu like symptoms for a couple days afterwards” “just sort of more general with vaccination injection things like having a sore arm or stiff arm”	10 (76.9)	0 (0.0)
	Concerned about getting a blood clot as a result of a vaccination	“The blood clot would be a big one” “I am sort of slightly worries about getting a brain clot”	4 (30.8)	3 (60.0)
	Vaccinations may impact fertility	“There hasn’t been a long enough time has passed to see the effects on fertility and effects on babies being born (…), and that would be a big concern of mine”	0 (0.0)	3 (60.0)
	Risk of side effects as a reason not to vaccinate	“I’m not willing to take that risk now” “I could die”	0 (0.0)	5 (100.0)
	Risk of side effects small enough to get vaccinated	“You know we can get blood clots from it, but the likelihood is so small” “The risk of blood clotting with the vaccination is that low, it’s very similar to the average blood clot statistics of the UK anyway”	13 (100.0)	0 (0.0)
	Concerned about the unknown long term effects of the COVID-19 vaccinations	“We won’t know that until a year, two years into looking at the side effects of the vaccine” “I think it could be long term risk, but we don’t know them yet, basically”	1 (7.7)	3 (60.0)
	Catching COVID-19 is worse than the side effects of vaccinations	“It’s worth the side effects for like two days” “I’m less worries about side effects because I think the side effects are better than getting COVID itself”	5 (38.5)	0 (0.0)
*Risk of not being*? *vaccinating*	Risk of not being vaccinated, is catching COVID-19.	“So obviously getting COVID” “I suppose if I don’t get vaccinated, I’m more prone to catching it”	6 (46.2)	3 (60.0)
	Risk of being excluded (social isolation)	“and you can’t go into certain places without being vaccinated, you can’t be settled in certain places, so it’s just segregation that is the main risk for not being vaccinated now” “So there’s a risk of social isolation”	7 (53.8)	5 (100.0)
	Being a risk to others	“I’m a potential harm for other people who are wither much older than me or immune compromised (…)” “I think the risk is passing it on to other people”	5 (38.5)	1 (20.0)
	Not being able to travel	“Will stop you from travelling (…)” “Also you won’t be allowed to travel”	7 (53.8)	5 (100.0)
	Risk of getting seriously ill	“High risk of death” “Potentially getting it myself and becoming more ill than I would be if I am, if I do get the vaccines”	5 (38.5)	2 (40.0)
	No consequences of not being vaccinated	“So basically no impact at all” “At the moment I work from home and I’ve only got my partner around so, really, it wouldn’t affect me too drastically”	2 (15.4)	1 (20.0)

The majority perceived it unlikely for them to contract COVID-19 (69.2% of intenders, 100% of non-intenders). No non-intenders believed it was likely for them to catch COVID-19, and only four intenders (30.1%) reported a high perceived susceptibility. Arguments for feeling unlikely in both groups were the decreasing infection rate in the UK (16.7%), taking necessary precautions (22.2%), and believing that because they did not contract it thus far means that they are unlikely to contract it now (27.8%). The majority (69.2%) of intenders perceived that their likelihood of catching COVID-19 would reduce after being vaccinated.

Regarding perceived risk of severe consequences of a COVID-19 infection, answers ranged from very low to very high risk. Nine intenders (69.2%) and four non-intenders (80.0%) reported a low perceived risk, while six intenders (45.2%) and one non-intender (20.0%) reported a high perceived risk. The most prevalent reasons were age and not having underlying health issues. Participants expected to be asymptomatic or experiencing only mild, flu-like symptoms. In addition, three intenders (23.1%) believed the vaccinations will reduce their chance of getting seriously ill from COVID-19, and three intenders (23.1%) worried about experiencing long-term consequences after a COVID-19 vaccination. Other risks mentioned in both groups were not being able to go to work (11.1% of total), infecting other people (5.6% of total), and reduced quality of life (5.6% of total).

In terms of risks of side effects, differences were observed between intenders and non-intenders. All intenders believed the risk of side effects was small enough to get vaccinated. On the contrary, all non-intenders reported that the risk of side effects was a reason not to get vaccinated. Despite not considering the side effects a major risk, all intenders showed awareness of potential side effects, both mild (e.g. slight fever, stiff arm, flu symptoms) and severe (blood clotting, seizures). Nine intenders (69.2%) expected only mild side effects, or no symptoms at all. Moreover, five intenders (38.5%) explicitly stated that potential side effects could not be as bad as catching COVID-19. The risk of blood clotting was addressed by participants of both groups. Four intenders (30.8%) reported worrying about getting a blood clot, however, it did convince them to remain unvaccinated. Three non-intenders (60.0%) were worried about blood clotting. Moreover, three non-intenders (60.0%) expressed concerns about the potential impact of the vaccinations on their fertility. This belief was not observed in the intenders. Finally, three non-intenders (60.0%) reported to worry about the potential long-term side effects of the vaccinations. Only one intender reported this (7.7%).

Finally, participants were asked what they considered risks of not being vaccinated. All participants, both intenders and non-intenders, reported that catching COVID-19 was one of the main risks. Moreover, most participants were worried about not being able to go to certain places in the future, when not being vaccinated. This belief was reported by all of the non-intenders and ten of the intenders (76.9%). In addition, frequently reoccurring was the ability to travel, as all non-intenders reported the concern of not being able to travel. The same belief was reported by seven intenders (53.8%). Five intenders (38.5%) reported that a risk of not being vaccinated was potentially infecting other people, which was also reported by one of the non-intenders (20.0%). Finally, two intenders (15.4%) reported that not being vaccinated would not have any consequences for them. This belief was expressed by one non-intender (20.0%).

### Motivation phase beliefs

Regarding the advantages, both groups reported similar examples of advantages. Nine intenders (69.2%) reported that being vaccinated would give them a certain sense of peace of mind. More specifically, eight intenders (61.5%) reported that it is an advantage that they are less likely to catch COVID-19. It was also stated by eight intenders (61.5%) that it is an advantage that they are less likely to infect other people. The former was not reported by the non-intenders, while one non-intender (20.0%) reported the latter. While all non-intenders reported that not being able to travel was one of the main risks of not being vaccinated, only two of them (40.0%) reported the ability to travel as potential advantage of being vaccinated. The ability to go out and socialize, on the contrary, was reported as a potential advantage by all of the non-intenders and seven intenders (53.8%). Three intenders (23.1%) reported that it is an advantage that the vaccinations will allow them to get back to normal life.

In term of disadvantages, all non-intenders reported that the disadvantages outweigh the advantages of being vaccinated. The disadvantages reported by this group referred to the potential risk of side effects and unknown long term effects, similar to the reported risks of being vaccinated in terms of side effects. Seven intenders (53.8%) reported that they did not see any disadvantages of being vaccinated, and only one (7.7%) reported to be uncertain about the long term effects. Only three intenders (23.1%) reported that the potential short term side effects were a disadvantage. Finally, it was reported by one intender (7.7%) that it could be a disadvantage that people do not comply to the other measures anymore as a result of being vaccinated, potentially resulting in another spike in infections.

When discussing affective advantages and disadvantages of being vaccinated, all intenders considered the vaccinations as important. On the contrary, the majority of the non-intenders (80.0%) thought the vaccinations were not important at all, and one (20.0%) reported that vaccinations could be important for others but not necessarily for themselves. Five intenders (38.5%) stated that they considered vaccinations important for the sake of protecting others rather than themselves. Seven intenders (53.8%) did not think the vaccinations are stopping people from getting the virus, which was also stated by three non-intenders (60.0%), and five intenders (38.5%) stated that they believed the vaccinations are effective in minimizing your response to an infection. Two non-intenders (40.0%) reported that the effectiveness of COVID-19 vaccinations was still unknown.

The construct of self-efficacy was divided in three sub-constructs: perceived difficulties, perceived accessibility and availability, and feeling sufficiently informed. Most participants reported that it was easy to obtain the vaccine. All non-intenders reported that if they wanted to get vaccinated, it would be easy to do so. Apart from one, all other intenders (92.3%) perceived the booking and scheduling system as straightforward. It was reported by three intenders (23.1%) that the main difficulty was waiting to become eligible for a vaccination, while three intenders (23.1%) believed they became eligible sooner than expected.

Even though none of the intenders believed the availability and accessibility to be a barrier in obtaining a vaccination, it was reported by six of them (46.2%) that it would be easier if the vaccination was provided in a different location and five of them (38.5%) reported that it would be easier if more time slots were available for appointments. Four intenders (30.8%) believed obtaining a vaccination was as easy as it could be. In terms of feeling sufficiently informed, all of the non-intenders believed crucial information was lacking, especially on side effects and long term effects. However, three of them (60.0%) stated that they knew enough to make their decision on the vaccinations. This belief was also observed in six intenders (46.2%). Furthermore, two intenders (15.4%) mentioned that they would like to know beforehand which vaccine they would receive, but this did not change their intention.

The final construct in the motivation phase was *social influence*, consisting of three sub-constructs; social norms, modelling, and support/pressure. Almost all participants reported to share the same opinion as their friends/family. Ten intenders (76.9%) reported that their social circle is positive towards vaccinations and three non-intenders (60.0%) reported that their friends and/or family were mostly negative towards vaccinations. Three intenders (23.1%) and two non-intenders (40.0%) stated that the opinions in their social circle vary between positive and negative. Moreover, three intenders (23.1%) stated that they believed that getting vaccinated is the right thing to do.

Six intenders (46.2%) stated that they do not believe the opinions of others are influencing their opinion. This was also stated by three non-intenders (60.0%). Three intenders (23.1%) addressed that they attach high value to the opinion of experts. This belief was also observed in two non-intenders (40.0%). Two intenders (15.4%) addressed that they believed the opinion of others was reinforcing their opinion. Finally, participants were asked who would support, or not support them in getting vaccinated. Five intenders (38.5%) believed that their friends and/or family would disapprove of them in case they did not get vaccinated. Although all non-intenders reported that (part of) their close social circle has negative attitudes towards vaccines, four of them (80.0%) also reported that there would be people in their environment who would disapprove of them not getting vaccinated. The motivation phase beliefs can be found in Table 3.

**Table 3:** Motivation phase beliefs.

Construct and sub-themes	Specific belief	Sample quotes	Frequency intenders (n = 13)	Frequency non-intenders (n = 5)
**Attitude (%)**				
***Perceived advantages***	Advantages of having a COVID-19 vaccination outweigh disadvantages	“I feel like the benefits far outweigh the negatives” “I’d say the disadvantages are always just lower than the advantages” “I feel the risk is outweighed by the benefits”	3 (23.1)	0 (0.0)
	Peace of mind resulting from being vaccinated	“Just a peace of mind, really” “A certain sense of peace of mind” “Just the peace of mind that I’m less likely to get COVID if I’m getting the vaccine”	9 (69.2)	0 (0.0)
	Less likely to contract COVID-19.	“but at least I know I have some level of protection in my body now” “I’m less likely to get COVID and pass it on to other people”	8 (61.5)	0 (0.0)
	A COVID-19 infection will not result in severe illness	“in order for in the future to not get put on a ventilator or be more unwell than you would be even if you didn’t get COVID that badly”	2 (15.4)	0 (0.0)
	Less likely to infect others with COVID-19 when being vaccinated	“Without the worry of infecting other people” “also so you don’t contract it, you don’t pass it on to people who are vulnerable and stuff like that”	8 (61.5)	1 (20.0)
	Ability to travel once vaccinated	“There is the fact that if I want to travel to certain places and a requirement is put in on proof of vaccination, I won’t be excluded”	5 (38.5)	2 (40.0)
	Ability to socialize once vaccinated	“So travel, and be able to socialize more freely (…)”	7 (53.8)	5 (100.0)
	Beneficial for the UK economy	“If everything started up again I might have more chance to get a job” “People will be able to go back to work again”	2 (15.4)	0 (0.0)
	Going back to normal life because of vaccinations	“I just want some of my pre COVID life back and I feel this is the biggest step in that direction” “May make us like return to normal life for once again”	3 (23.1)	0 (0.0)
***Disadvantages***	Disadvantages of having a COVID-19 vaccination outweigh advantages	“There are more negatives than positives to this”	0 (0.0)	5 (100.0)
	No perceived disadvantages of being vaccinated	“I don’t really see any disadvantages …” “I can’t name any disadvantages of getting a vaccination”	7 (53.8)	0 (0.0)
	Potential short term side effects	“Feeling ill for the days following it, though I’ve decided that it’s a price I’m willing to pay to be vaccinated. So that is a disadvantage”	3 (23.1)	0 (0.0)
	Unknown long term side effects	“There’s not really any long term studies, like scientists are just watching as we go along and to see if there’s any long term risk, so I believe that’s the only disadvantage”	1 (7.7)	3 (60.0)
	People not complying to the other measures anymore	“I think that what I was worried about when the vaccines were started rolling out is that people would get very confident and think, OK well, I have the vaccine, so I’ll just be able to go out and will be able to go to clubs and things like that”	1 (7.7)	0 (0.0)
***Perceived effectiveness***	Not stopping you from getting the virus	“I do not think the vaccine will stop you from getting the virus” “It doesn’t stop you from catching COVID (…)”	7 (53.8)	3 (60.0)
	Minimizing your response to an infection.	“I think it minimizes your response to it” “(…) since we’ve been vaccinating far far far less people are needing to go to hospital (…)”	5 (38.5)	0 (0.0)
	Unknown effectiveness of COVID-19 vaccinations	“I think only time’s gonna tell” “I don’t really think anybody knows yet”	0 (0.0)	2 (40.0)
***Perceived importance***	COVID-19 vaccinations perceived as important	“I believe the vaccines are extremely important” “I think it is very important just to stop the infection rate from growing”	13 (100.0)	0 (0.0)
	The only way to can end the pandemic.	“There doesn’t seem to be any other solution” “They are probably the only way that we can actually get out of this pandemic”	8 (61.5)	0 (0.0)
	Everybody should get vaccinated	“regarding the wider public, I think everyone should” “Very important. I think everybody should get vaccinated”	2 (15.4)	0 (0.0)
	Important to get vaccinated for the sake of others	“I think for the elderly more than anything” “Like for myself, no, but for the others around me”	5 (38.5)	1 (20.0)
	No perceived importance	“I don’t think they are important at all” “I just think if it was that important to curbing COVID cases and protecting people, then all these extra measures wouldn’t need to be taken. So no, I don’t think the vaccinations are as important as we may feel there”	0 (0.0)	4 (80.0)
**Self-efficacy (%)**				
***Perceived difficulties in obtaining the vaccination***	Difficult to book an appointment	“The booking system hasn’t been very great”	1 (7.7)	n/a
	No practical difficulties in obtaining a vaccination	“I don’t think there are any problems” “No, I can’t think of anything else”	11 (84.6)	4 (80.0)
	Need for more timeslots available to get vaccinated	“Limited time options for second dose” “More appointment slots I think. Because when I received the text there was only one day available and obviously more days would have enabled me to probably work and get the vaccine”	5 (38.5)	n/a
	Waiting for eligibility	“It took time” “The biggest difficulty that I had was just waiting for, you know, my age group to be eligible”	3 (23.1)	0 (0.0)
	Need for more/different vaccination locations	“Not easy for myself, so I don’t drive, so I need somebody to drive over to the vaccine centre, but I feel it would be easier if I lived in a different place, for example somewhere like London, where there’s good public transport, (…)”	6 (46.2)	n/a
***Perceived capability–accessibility and availability***	Easy to book an appointment	“It was easy to book into the system to get a COVID vaccination” “I just had it through the post and I didn’t have to ask or anything, it just came very easy”	4 (30.8)	n/a
	Vaccine centres are easily accessible	“There was a site on my university campus which was about 10-minute walk” “(…), I feel like fairly well catered to in terms of availability and local position I guess”	13 (100.0)	0 (0.0)
	Getting vaccinated could not be easier as it is	“I think it’s as easy as it could have been for me in my case” “I think it’s as easy as it could be”	4 (30.8)	0 (0.0)
	Eligible earlier than expected	“No, it was really quick and much quicker than I thought” “I did expect it to have it like 3 or 4 months later than I did so I wasn’t really even thinking about it. And then it came through so I was quite pleased”	3 (23.1)	0 (0.0)
***Feeling sufficiently informed***	Informed enough to make a decision	“I would say it’s sufficient” “I am more than informed to make that choice and hopefully it stays that way. So I think I’m primarily informed enough to make that decision and choice if need be”	6 (46.2)	3 (60.0)
	Crucial information is lacking	“I’d rather see a long term thing and then be able to make a more informed decision than without, just jumping into something because I like to do research and things like that”	3 (23.1)	5 (100.0)
**Social influence (%)**				
***Modelling***	Friends/family all not getting vaccinated	“No one else in my circle is pro vaccine, nor my family” “My immediate circle do not really, are not going to get the vaccine, do not really think about it, and are really terrified of it”	0 (0.0)	3 (60.0)
	Friends/family all getting vaccinated	“Most of my friends get vaccinated (…)” “Some of them who are my age have also been vaccinated, so I am keen to get vaccinated as well”	10 (76.9)	0 (0.0)
	Mixed opinions within social circle	“It is really mixed, it’s literally a 50/50 split” “It’s a bit sort of hit a mess”	3 (23.1)	2 (40.0)
***Social norms***	Getting vaccinated is the right thing to do	“The expectation is that when you have the opportunity to get vaccinated, you will get vaccinated, because that’s the right thing to do”	3 (23.1)	0 (0.0)
	Expert opinions are most important	“The biggest opinion for me was from people who work in healthcare or in the science, and they sort of gave me the opinion that the vaccine would have been rigorously tested and its safe, that was just it for me”	3 (23.1)	2 (40.0)
	Opinions of other are not influencing personal opinion	“Not really, I think. My mind is pretty made up to be honest” “No, I wouldn’t let that impact me”	6 (46.2)	3 (60.0)
	Opinions of others reinforce own beliefs	“It probably swayed me towards one or the other, but they’ve all gotten it, so maybe that swayed me a little bit more towards getting it”	2 (15.4)	0 (0.0)
***Support and Pressure***	People non supportive of the decision not to vaccinate	“So if I chose not to, then that would make me socially ostracized” “I don’t think my friends would be very happy and potentially would also be very angry with me if I decided not to have the vaccine”	5 (38.5)	4 (80.0)
	Support from close social circle	“They’re all positive. All of my friends have had their first jab and are making the appointment to get the second one” “My social circle is taking it well”	10 (76.9)	5 (100.0)
	People discouraging to vaccine	“I think my dad is against it” “My mom was actually very against the idea of getting a vaccination, but she is one of those people who would see things on WhatsApp quite a lot and would be ‘oh this was happening’”	5 (38.5)	4 (80.0)

### Action phase beliefs

Five participants (27.8% of total) reported not to intend to vaccinate at all. The participants were asked whether they would accept a vaccination regardless of the manufacturer. Four intenders (30.8%) reported doubts about the Astra Zeneca vaccine. Three of them (23.1%) would refuse the Astra Zeneca vaccine and one (7.7%) reported to only accept it when there was no other option. Two intenders (15.4%) did not book their appointment yet, all other intenders (84.6%) had either booked their appointment or had received their first dose. One participant (5.6% of total) reported to be fully vaccinated at the time of interviewing. Ten intenders (76.9%) did not prepare themselves in any specific way. Only three (23.1%) stated that they prepared themselves in case side effects occurred. For intenders, difficulties of getting vaccinated were discussed as part of the construct of self-efficacy, and participants were asked how they would cope with the named difficulties. Five intenders (38.5%) that expected side effects to occur, reported not to have a specific coping plan. Making travel arrangements was named as a coping strategy when vaccine centres were not accessible. For non-intenders, difficulties may arise as a result of not being vaccinated. Three non-intenders (60.0%) stated that if not being vaccinated would hold them back from travelling or going to certain places, they would try to avoid these places. Finally, two intenders (15.4%) and two non-intenders (40.0%) stated that they do not have a specific plan in how to cope with difficulties in case they arise. The action phase beliefs are presented in Table 4.

**Table 4:** Action phase beliefs.

Construct and sub-themes	Specific belief	Sample quotes	Frequency intenders (n = 13)	Frequency non-intenders (n = 5)
**Action planning (%)**	Accepting regardless of manufacturer	“Yes, if it’s Astra Zeneca, I’m not going to lie, I will be a little bit more nervous but no so nervous that I will refuse it” “I would accept any that was offered to me, because if it’s being offered to be then it should be relatively safe enough, if it’s offered by the NHS”	10 (76.9)	0 (0.0)
	Only accepting Pfizer or Moderna vaccines	“I wouldn’t accept the Oxford Astra Zeneca vaccine, but I would accept a Pfizer or Moderna vaccine” “But if they offer me Astra Zeneca probably I would refuse”	3 (23.1)	0 (0.0)
	Not accepting any of the vaccines	“At this point in time, for me, no, I’m just sticking to the usual methods of social distancing and trying to just be cautious in public situations, I’d say” “I don’t intend to accept it at all”	0 (0.0)	5 (100.0)
**Preparatory planning (%)**	Not preparing for getting vaccinated	“I’m just gonna turn up, I guess it’s not real preparation for me” “I won’t do anything in particular”	10 (76.9)	n/a
	Preparing for potentially experiencing side effects	“We’ve agreed that she will basically keep her schedule open as much as possible in the days following my vaccination, so if I do feel ill, she’ll come look after me”	3 (23.1)	n/a
**Coping planning (%)**	Not taking action in case side effects occur	“I’ll just stay in bed for a couple of days. Really, I am not worried.” “If I do have any side effects, then I’ll take the time off work (…)”	5 (38.5)	n/a
	Avoiding places where being vaccinated is mandatory	“My plan was just boycott everywhere that asks because really we can’t have freedom of choice over our own bodies and I don’t want to give money to anybody” “(…) or maybe find countries that are lax about getting vaccinations or not”	n/a	3 (60.0)
	No specific coping plans in case of experiencing difficulties	“I’ll figure out as I go along, but I cannot say anything for definite now” “I’m gonna see what happens if it becomes mandatory that you have to have vaccine, I may consider it, but I’m definitely not going to have it just because you have to” “I haven’t really thought about it to be honest”	2 (15.4)	2 (40.0)

## Discussion

This study aimed to identify and understand the beliefs young adults have about COVID-19 vaccinations in the UK by applying the I-Change Model. A comparison was made between the beliefs of participants indenting to vaccinate and participants not intending to vaccinate. Intenders and non-intenders did not differ from each other in terms of knowledge. However, the most frequently mentioned source of information by both groups was the news, while Murphy et al. [13] concluded that non-intenders were less likely to use traditional or authoritative sources as sources of information. The research by Murphy et al. [13] was however conducted on the general UK population, not stratifying for different age groups and cannot be directly compared to the target group of the present study.

Noteworthy is that there are also beliefs identified in previous research that were not observed in the present study. Research conducted by Freeman et al. [11] and Murphy et al. [13] showed that a significant minority of people unwilling to vaccinate held general vaccine conspiracy beliefs and negative perceptions of healthcare professionals, and negative healthcare experiences. However, none of these beliefs were observed in the present study population, which may indicate that these topics are less relevant for young adults.

In terms of risk perception, both groups demonstrated similar beliefs on perceived susceptibility and severity of COVID-19, with most participants only reporting a low perceived risk. These findings are in line with the findings of the Office for National Statistics [12] and Sherman et al [19], who reported that unwillingness to vaccinate was associated with a low perceived susceptibility and severity [19, 27]. The main difference between the two groups was observed considering beliefs on risk of side effects. The perceived risk of side effects and unknown (long-term) effects, such as risk of blood clotting and potentially affecting fertility, was a reason not to get vaccinated for all of the non-intenders. This is in line with prior studies, such as by the OFS [12], Sherman et al. [19] and Freeman et al. [11], which concluded that the primary concerns of people not wanting a COVID-19 vaccine were not trusting the safety of the vaccines and the belief that there is not enough long term evidence available yet. While the present study did not focus on differences in beliefs between different genders, Umakanthan & Lawrence [10] found that being a woman is negatively associated with willingness to vaccinate. This finding may be explained by the belief that the vaccines are potentially affecting fertility. Further research is needed to determine if a relationship between these findings exists. The findings regarding safety however suggest that in communication interventions, positive information regarding vaccination should be used rigorously, as suggested by Umakanthan et al. [28].

As part of the motivation phase, the intenders mostly could not name any disadvantages. For the non-intenders, the disadvantages outweighed the perceived advantages, mostly referring to the risk of unknown effects and risks, which was in line with the findings in the risk awareness phase as well as prior research [11, 19, 27, 29]. Potential advantages were the ability to travel, ability to socialize, and reduced susceptibility and severity of an infection. As the vaccination roll-out was still in process at the time of the study, and participants were unsure about the consequences of not being vaccinated on their ability to socialize and to travel, it would be valuable to investigate whether the intention to vaccinate chances when such restrictions are enforced.

Considering social influence, most participants shared the same beliefs as their friends and/or family, which could indicate that the social circle can be both a facilitator in case of positive attitudes and a barrier in case of negative attitudes. However, most participants stated that they do not think that beliefs of others influence theirs. This could be related to the findings of Sinclair & Agerström [30], who concluded that the effect of signalling social norms on vaccine acceptance is only limited. Further research could investigate whether there is a causal relationship between personal beliefs and beliefs of others. In terms of self-efficacy, availability and accessibility were not perceived as a barrier in both groups. However, a lack of information and feeling insufficiently informed can be considered as barriers for the non-intenders. This is in line with the findings from the risk perception and disadvantages constructs as well as the findings of the aforementioned studies.

In the action phase, intenders mostly did not deem it necessary to prepare themselves in any way, nor had specific coping plans in case difficulties would arise. Moreover, when non-intenders were asked about coping strategies as to deal with difficulties of not being vaccinated, avoidance behaviour was most prevalent. In addition, most participants did not foresee any difficulties and thus did not consider coping strategies. Some stated that regardless of difficulties, they would still proceed with fulfilling their intention to either get vaccinated or refuse a vaccination, demonstrating stability of intentions. Behaviours related to coping planning, to the best of our knowledge, not been investigated in other research yet, therefore providing new insights.

This study showed that the main reason for not intending to vaccinate among young adults, is the risk of (long term) side effects and the lack of evidence on long term consequences of getting vaccinated. Even though non-intenders were aware of the potential benefits of being vaccinated, their perceived disadvantages still outweighed the advantages. However, current COVID-19 vaccination promotion campaigns, both targeting the general population as well as the young adult population of the UK, are mostly focussing on the benefits of being vaccinated. The promoted benefits include being protected, protecting others, and being able to go back to normality [31]. Interestingly, these benefits seem to be not convincing enough for non-intending young adults. Therefore, it is recommended for health communication interventions to focus on taking away as much uncertainty as possible and to be transparent, ensuring availability of evidence for the entire public and focusing on the safety of the vaccines.

This study explored the difference in underlying beliefs regarding intention to vaccinate among young adults, comparing intenders and non-intenders. Understanding why people do or do not engage in certain behaviour is crucial for being able to change this particular behaviour. As explained by Kok et al. [32], behaviour change methods usually target determinants of behaviour. However, determinants of behaviour are usually aggregates of specific beliefs and may therefore be too general to target directly. Thus, in order to develop tailored interventions, it is necessary to identify the underlying beliefs of the determinants of behaviour, as suggested in the pragmatic approach of Cheung et al. [17] in designing tailored digital health interventions. Currently, literature only focuses on the determinants of vaccination behaviour. This study now adds to the body of literature what specific beliefs should be targeted when creating tailored interventions in which the health messages are personalised to the individual beliefs of the target population.

### Limitations

As the COVID-19 pandemic is a rapidly changing situation with new findings emerging every single day, one of the main limitations of this study is that it is only a snapshot in time. Beliefs regarding the COVID-19 vaccinations are likely to change over time due to external influences. This may cause participants to change from non-intender to intender, or vice versa, making the dependent variable of this study unstable. Moreover, intention to vaccinate may not result in the action of getting vaccinated. Therefore, the results have to be interpreted with caution. A quantitative follow-up study, preferably a longitudinal cohort study, could further explain the correlation between beliefs and intention to vaccinate, as well as the changes in beliefs over time.

The study is dependent on self-reported data by the individuals, who may be biased in their views on the COVID-19 vaccination This may potentially result in people expressing the intention to vaccinate (without being vaccinate yet) as this is considered a socially desirable answer. The interviewer and moderator made an effort to make the participant feel at ease and comfortable in speaking freely about their opinions and beliefs in order to avoid social desirability bias.

People who have received one or more doses of a COVID-19 vaccine or expressing that they are still planning on getting vaccinated against COVID-19 were coded as people intending to vaccinate. People expressing that they did not get vaccinated against COVID-19 and are not planning to do so were coded as people not intending to vaccinate. If the dichotomisation of the dependent variable was based on different coding, slightly different frequencies between intenders and non-intenders may have occurred. However, the content of the variety of perceptions would most likely not have changed. Moreover, the purpose of this paper was not to capture the true prevalence of intenders and non-intenders, but to gain understanding about potential differences between the two groups in terms of their beliefs regarding COVID-19 vaccinations.

Furthermore, to be able to compare intending and non-intending individuals, it was necessary to apply a purposive sampling technique. The participants included in this study may differ in their attitudes and behaviours from individuals who did not participate in this study as a result of their willingness to participate in scientific research through their Prolific membership. Moreover, it is possible that preliminary data saturation has occurred as a result of the recruitment methods applied. Due to the limited sample size, the findings are not generalizable to the UK general population. Therefore, further research on a larger, representative study population, is necessary to quantify the findings of this study and to get statistically significant results.

## Conclusion

This study revealed that young UK adults differ in their perceived risk of side effects and attach a different weight to the advantages of being vaccinated. Moreover, most individuals, both intending and non-intending, report that their personal opinions are similar to their social environment. In terms of action, both groups do not report clear preparation or coping plans. The results of this study can be used to tailor health communication to the needs and preferences of the target population, resulting in more effective campaigns and a higher vaccination rate among young adults. This study is a first step in exploring the salient beliefs regarding COVID-19 vaccinations in young adults in the UK. A quantitative follow-up study, preferably a longitudinal cohort study, could further explain the correlation between beliefs and intention to vaccinate.

## Supporting information

S1 File Interview guide. (DOCX)

S2 File Coding guide. (DOCX)

S3 File Inclusivity in global research questionnaire. (DOCX)

S1 Fig Awareness phase beliefs. (TIF)

S2 Fig Motivation phase beliefs. (TIF)

S3 Fig Action phase beliefs. (TIF)
