# Supplementary material for: Beliefs regarding COVID-19 vaccinations of young adults in the United Kingdom: An interview study applying the Integrated Change Model
Source: PLoS One. 2022 Dec 6;17(12):e0277109. doi: 10.1371/journal.pone.0277109 (PMC9725152; doi:10.1371/journal.pone.0277109)
Supplement: S1 File — (DOCX) [file pone.0277109.s001.docx]

# S1. Interview guide

**Introduction and consent**

Dear participant,

Thank you for taking the time to participate in our study.

In a few minutes, the interview will start. The general aim of this study is to identify the beliefs that can be drivers or barriers to obtaining a COVID-19 vaccination and to discover preferences regarding COVID-19 vaccinations. The interview consists of three parts. We will start with some general demographic questions. Then we will go into detail about your COVID-19 vaccination perceptions. Lastly, we will ask you to rank several attributes based on their importance. Please try to answer based on your first intuition. There are no right or wrong answers. Please feel free to ask clarification if questions are unclear to you. If you wish not to respond to a question, feel free to inform us. Your answers will be processed anonymously.

In order to process your answers as accurately as possible, we will record the audio of the interview. We will ask for your permission to record twice: once before starting the recording and once while recording. The audio tapes will be erased after transcribing the interviews.

*ask participant:*

- Do you have any questions before starting the interview?
- Do you agree to participate in this study?

*ask participant:*

- Do you give us permission to record the audio of the interview?

***** start recording *

*ask participant:*

- Do you give us permission to record the audio of the interview?
- Do you agree to participate in this study?

**Demographics**

1. What is your age?
2. What gender do you identify yourself with?
   - Female
   - Male
   - Other
3. In which region in the UK do you reside?
   - Scotland
   - Northern Ireland
   - Wales
   - North East
   - North West
   - Yorkshire and the Humber
   - West Midlands
   - East Midlands
   - South West
   - South East
   - East of England
   - Greater London
4. What is your highest level of education completed?
   - Degree
   - A level or equivalent
   - GCSE or equivalent
   - Other qualification
   - No qualification
5. What is your current employment status?
   - Student
   - Unemployed – voluntarily
   - Unemployed – searching for a job
   - Unemployed – medical reason
   - Part-time employed
   - Full-time employed
   - Other
6. What is your current vaccination status?
   - Vaccinated
   - Not vaccinated (refused vaccination)
   - Not vaccinated (waiting for invitation)
   - No comment

**Questions beliefs**

**Awareness**

Knowledge:

1. What do you know about COVID-19 vaccinations and vaccinations in general?

(Prompt: Think of eligibility, manufacturers, safety, etc.) What is your main source of information?

Risk perception:

1. How likely do you think it is that you will contract COVID-19?
2. What are/could be the risks of contracting COVID-19?

(Prompt: For yourself? For your family?)

1. What are/could be the risks if you decide to get vaccinated against COVID-19?

(Prompt: For yourself? For your family? What are potential harms? i.e. side effects, health risks, confidence in safety)

1. What are/could be the risks if you do not get vaccinated against COVID-19?

(Prompt: For yourself? For your family? i.e. regarding health, work, social life?)

**Motivation**

Attitude:

1. What is/would be the advantages for you to receive a COVID-19 vaccination?

(Prompts: What are the advantages? - Regarding health? Work? Social life?)

1. What are/could be the disadvantages of receiving a COVID-19 vaccination?

(Prompts: Regarding health? Work? Social life?)

1. How important do you think vaccinations are in stopping the COVID-19 pandemic?

(Prompt: Who do you think they are important for? Why are they (not) important?)

Social influence:

1. How is your social circle perceiving the COVID-19 vaccinations? / Would anyone in your social cycle influence whether or not you want to get a COVID-19 vaccination? How might the views or opinions of others affect your decision about getting a COVID-19 vaccination?

(Prompts: Who would support you? Who would be against you? Who already received/refused a vaccination?)

1. Is obtaining a COVID-19 vaccination something you feel you need to do?

(Prompt: for who? why so?)

Self-efficacy:

1. Do you feel like you are informed enough about the COVID-19 vaccines?
2. Are there any factors making it difficult for you to obtain a COVID-19 vaccination?
3. Are there any factors making it easy (/easier) for you to obtain a COVID-19 vaccination?

**Action**

Intention:

1. When a COVID-19 vaccine becomes available for you (regardless of the manufacturer), do you intend to accept it? (Confidence in vaccines, safety)

Preparatory planning:

1. How are you preparing yourself for getting a COVID-19 vaccination when it becomes available to you?

Coping planning:

1. What problems/difficulties do you think you might face when you are going to get your COVID-19 vaccination?

(Prompts: location of the vaccination, opinion of others, planning, side effects)

1. How are you planning to deal with difficulties arising when you are getting a COVID-19 vaccination?

(Prompt: What would help you to overcome these problems/difficulties?)

**Closing the interview**

1. Is there anything you would like to add yourself that has not been discussed yet, or are there any questions from your side?
