# Supplementary material for: Beliefs regarding COVID-19 vaccinations of young adults in the United Kingdom: An interview study applying the Integrated Change Model
Source: PLoS One. 2022 Dec 6;17(12):e0277109. doi: 10.1371/journal.pone.0277109 (PMC9725152; doi:10.1371/journal.pone.0277109)
Supplement: S2 File — (DOCX) [file pone.0277109.s002.docx]

# S2. Coding guide

| Phase | Construct | Sub-construct | Sub-themes |
| --- | --- | --- | --- |
| Awareness | Knowledge |  | Eligibility |
|  |  |  | Purpose |
|  |  |  | Manufacturers |
|  |  |  | Other |
|  | Risk perceptions | Susceptibility | Susceptibility to COVID-19 |
|  |  |  | Risk of side effects |
|  |  | Severity | Severity of COVID-19 |
|  |  |  | Perceived risk of not being vaccinated |
| Motivation | Attitude | Advantages | Advantages of being vaccinated |
|  |  |  | Perceived effectiveness |
|  |  |  | Perceived importance |
|  |  | Disadvantages |  |
|  | Self-efficacy | Perceived capability | Accessibility and availability |
|  |  |  | Feeling informed |
|  |  | Perceived difficulties |  |
|  | Social influence | Modelling |  |
|  |  | Social norms |  |
|  |  | Support | Positive attitudes |
|  |  |  | Negative attitudes |
| Action | Intention |  |  |
|  | Preparatory planning |  |  |
|  | Coping planning |  |  |
|  | Plan enactment |  |  |
