# Supplementary figures and images for: Beliefs regarding COVID-19 vaccinations of young adults in the United Kingdom: An interview study applying the Integrated Change Model
Source: PLoS One. 2022 Dec 6;17(12):e0277109. doi: 10.1371/journal.pone.0277109 (PMC9725152; doi:10.1371/journal.pone.0277109)

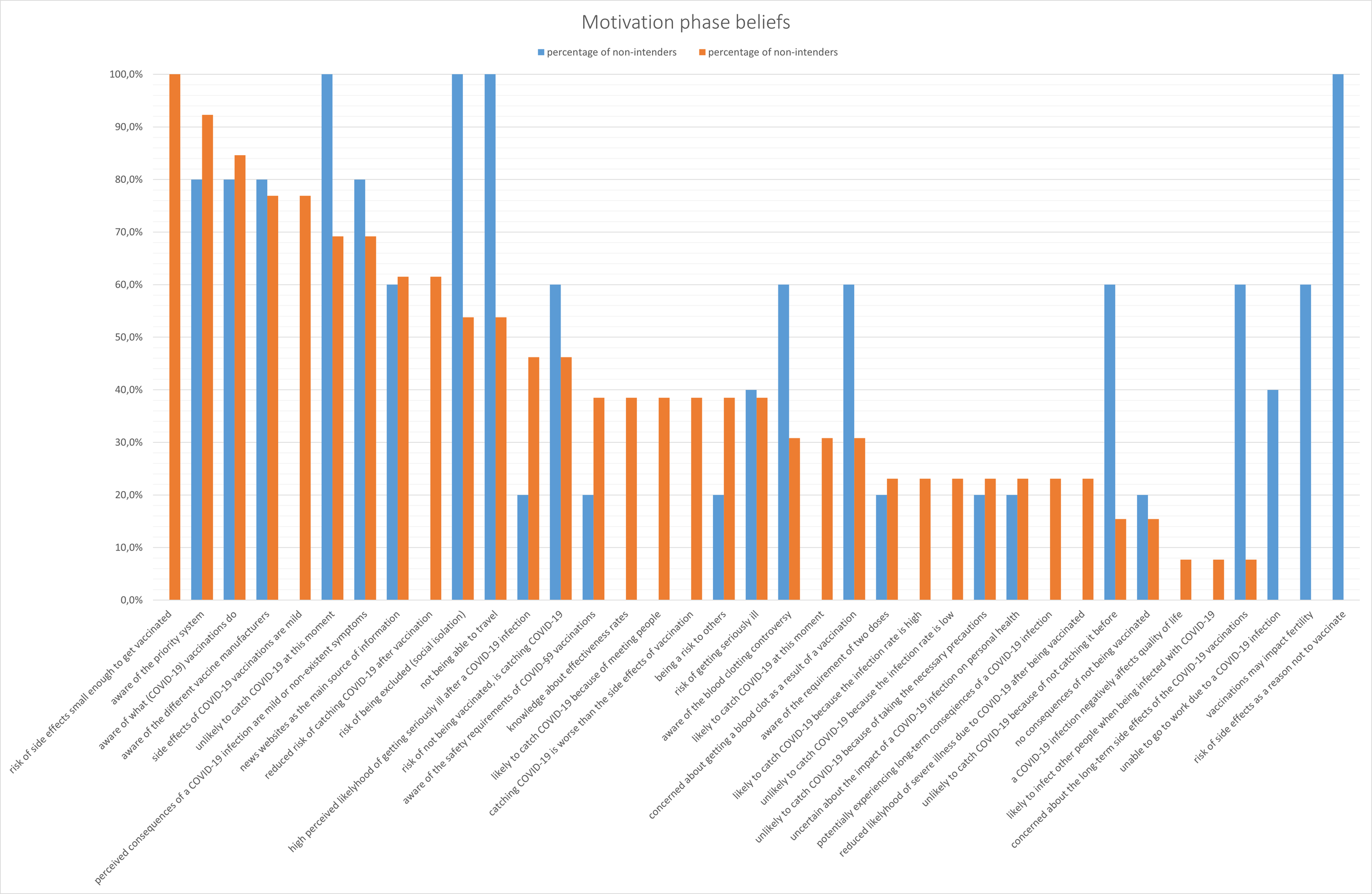

Supplement: S1 Fig — (TIF) [file pone.0277109.s004.tif]

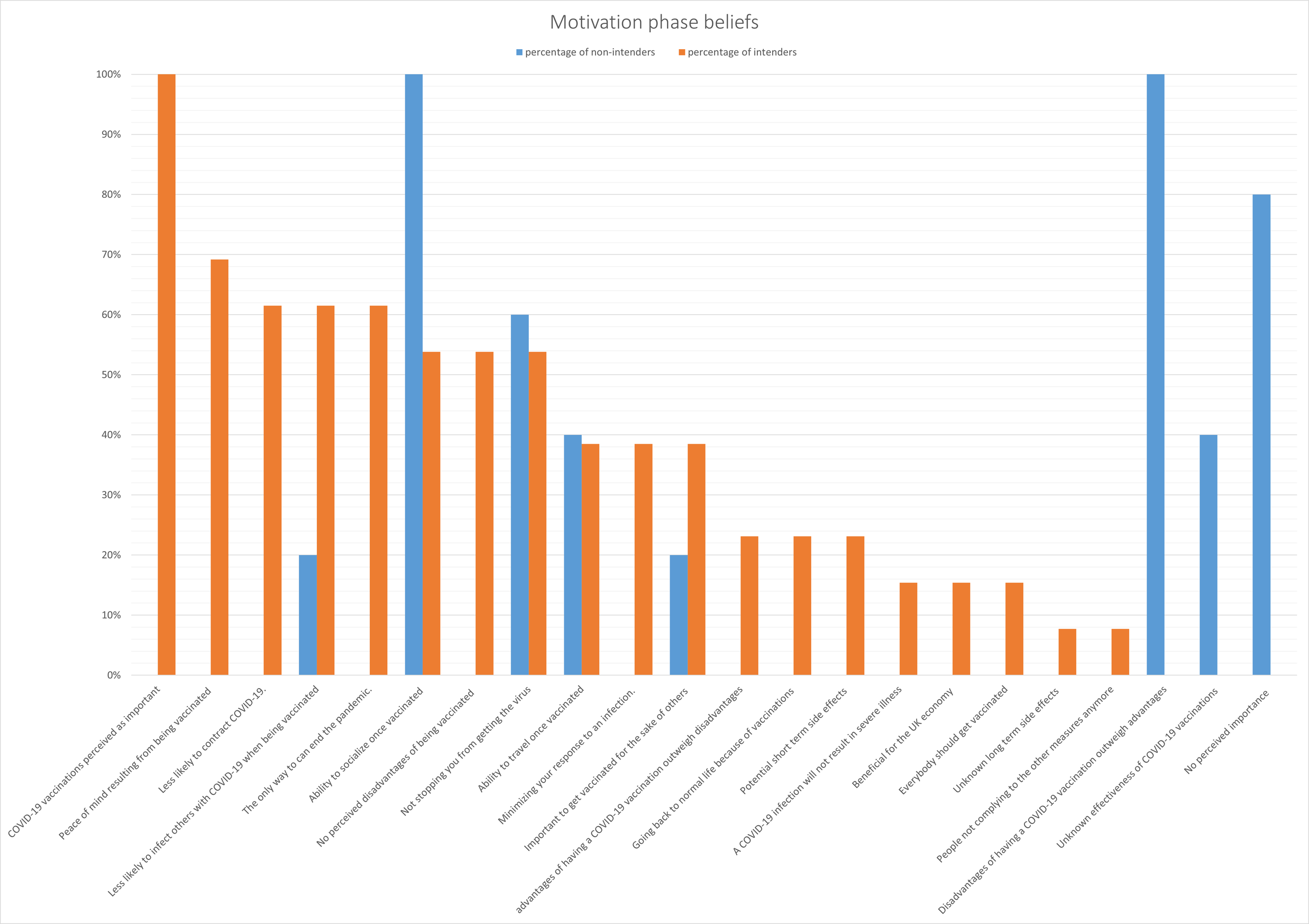

Supplement: S2 Fig — (TIF) [file pone.0277109.s005.tif]

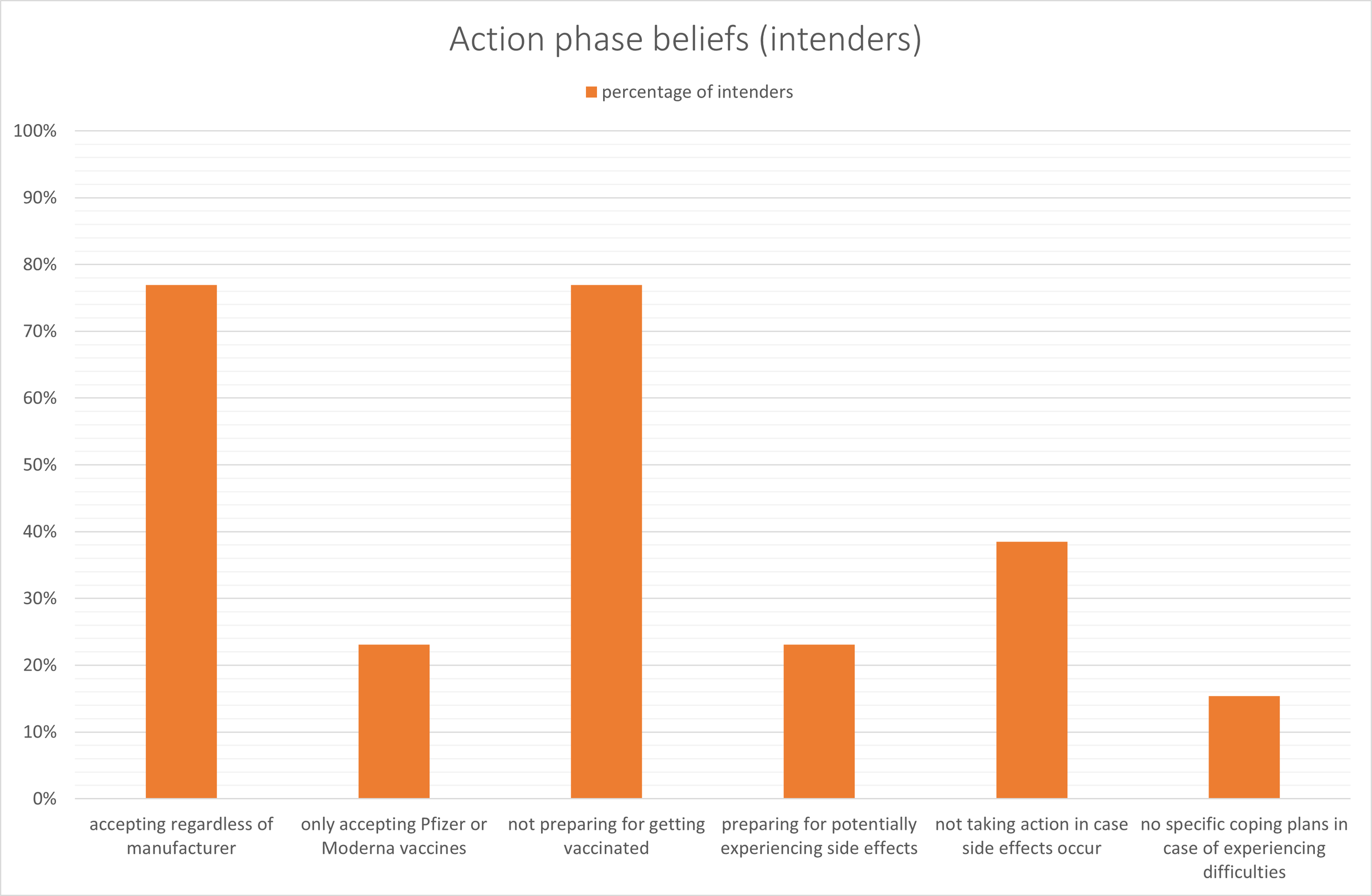

Supplement: S3 Fig — (TIF) [file pone.0277109.s006.tif]
